# Tobacco Use Among Middle and High School Students — United States, 2011–2014

**Published:** 2015-04-17

**Authors:** René A. Arrazola, Tushar Singh, Catherine G. Corey, Corinne G. Husten, Linda J. Neff, Benjamin J. Apelberg, Rebecca E. Bunnell, Conrad J. Choiniere, Brian A. King, Shanna Cox, Tim McAfee, Ralph S. Caraballo

**Affiliations:** 1 Office on Smoking and Health, National Center for Chronic Disease Prevention and Health Promotion, CDC; 2 Epidemic Intelligence Service, CDC; 3 Center for Tobacco Products, Food and Drug Administration

Tobacco use and addiction most often begin during youth and young adulthood (1,2). Youth use of tobacco in any form is unsafe (1). To determine the prevalence and trends of current (past 30-day) use of nine tobacco products (cigarettes, cigars, smokeless tobacco, e-cigarettes, hookahs, tobacco pipes, snus, dissolvable tobacco, and bidis) among U.S. middle (grades 6–8) and high school (grades 9–12) students, CDC and the Food and Drug Administration (FDA) analyzed data from the 2011–2014 National Youth Tobacco Surveys (NYTS). In 2014, e-cigarettes were the most commonly used tobacco product among middle (3.9%) and high (13.4%) school students. Between 2011 and 2014, statistically significant increases were observed among these students for current use of both e-cigarettes and hookahs (p<0.05), while decreases were observed for current use of more traditional products, such as cigarettes and cigars, resulting in no change in overall tobacco use. Consequently, 4.6 million middle and high school students continue to be exposed to harmful tobacco product constituents, including nicotine. Nicotine exposure during adolescence, a critical window for brain development, might have lasting adverse consequences for brain development (1), causes addiction (3), and might lead to sustained tobacco use. For this reason, comprehensive and sustained strategies are needed to prevent and reduce the use of all tobacco products among youths in the United States.

NYTS is a cross-sectional, school-based, self-administered, pencil-and-paper questionnaire administered to U.S. middle and high school students. Information is collected on tobacco control outcome indicators to monitor the impact of comprehensive tobacco control policies and strategies (4) and inform FDA’s regulatory actions (5). A three-stage cluster sampling procedure was used to generate a nationally representative sample of U.S. students who attend public and private schools in grades 6–12. This report includes data from 4 years of NYTS (2011–2014), using an updated definition of current tobacco use that excludes kreteks (sometimes referred to as clove cigarettes).* Of 258 schools selected for the 2014 NYTS, 207 (80.2%) participated, with a sample of 22,007 (91.4%) among 24,084 eligible students; the overall response rate was 73.3%. Sample sizes and overall response rates for 2011, 2012, and 2013 were 18,866 (72.7%), 24,658 (73.6%), and 18,406 (67.8%), respectively. Participants were asked about current (past 30-day) use of cigarettes, cigars (defined as cigars, cigarillos, or little cigars), smokeless tobacco (defined as chewing tobacco, snuff, or dip), e-cigarettes,† hookahs,§ tobacco pipes (pipes),¶ snus, dissolvable tobacco (dissolvables), and bidis. Current use for each product was defined as using a product on ≥1 day during the past 30 days. Tobacco use was categorized as “any tobacco product use,” defined as use of one or more tobacco products and “≥2 tobacco product use,” defined as use of two or more tobacco products. Data were weighted to account for the complex survey design and adjusted for nonresponse; national prevalence estimates with 95% confidence intervals and population estimates rounded down to the nearest 10,000 were computed. Estimates for current use in 2014 are presented for any tobacco use, use of ≥2 tobacco products, and use of each tobacco product, by selected demographics for each school level (high and middle). Orthogonal polynomials were used with logistic regression analysis to examine trends from 2011 to 2014 in any tobacco use, use of ≥2 tobacco products, and use of each tobacco product by school level, controlling for grade, race/ethnicity, and sex and simultaneously assessing for linear and nonlinear trends.** A p-value <0.05 was considered statistically significant. SAS-Callable SUDAAN was used for analysis.

In 2014, a total of 24.6% of high school students reported current use of a tobacco product, including 12.7% who reported current use of ≥2 tobacco products. Among all high school students, e-cigarettes (13.4%) were the most common tobacco products used, followed by hookahs (9.4%), cigarettes (9.2%), cigars (8.2%), smokeless tobacco (5.5%), snus (1.9%), pipes (1.5%), bidis (0.9%), and dissolvables (0.6%) (Table). Among high school non-Hispanic whites, Hispanics,†† and persons of non-Hispanic other races, e-cigarettes were the most used product, whereas among non-Hispanic blacks, cigars were used most commonly. Current use of any tobacco and ≥2 tobacco products among middle school students was 7.7% and 3.1%, respectively. E-cigarettes (3.9%) were the tobacco product used most commonly by middle school students, followed by hookahs (2.5%), cigarettes (2.5%), cigars (1.9%), smokeless tobacco (1.6%), pipes (0.6%), bidis (0.5%), snus (0.5%), and dissolvables (0.3%).

From 2011 to 2014, statistically significant nonlinear increases were observed among high school students for current e-cigarette (1.5% to 13.4%) and hookah (4.1% to 9.4%) use (Figure 1). Statistically significant linear decreases were observed for current cigarette (15.8% to 9.2%) and snus (2.9% to 1.9%) use. Statistically significant nonlinear decreases were observed for current cigar (11.6% to 8.2%), pipe (4.0% to 1.5%), and bidi (2.0% to 0.9%) use. Current use of any tobacco product (24.2% to 24.6%) and use of ≥2 tobacco products (12.5% to 12.7%) did not change significantly from 2011 to 2014. Among middle school students, similar trends were observed during 2011–2014 (Figure 2). A statistically significant linear decrease was observed only in middle school students currently using ≥2 tobacco products (3.8% to 3.1%).

In 2014, an estimated 4.6 million middle and high school students currently used any tobacco product, of which an estimated 2.2 million students currently used ≥2 tobacco products. Of current tobacco users, 2.4 million used e-cigarettes and 1.6 million used hookahs. The largest increase in current e-cigarette use occurred from 2013 to 2014. Current e-cigarette use tripled from 2013 (660,000 [4.5%]) to 2014 (2 million [13.4%]) among high school students (Figure 1); and among middle school students, prevalence increased by a similar magnitude, from 1.1% (120,000) to 3.9% (450,000) (Figure 2). From 2013 to 2014, substantial increases also were observed for current hookah use, with prevalence almost doubling for high school students from 5.2% (770,000) to 9.4% (1.3 million) and for middle school students from 1.1% (120,000) to 2.5% (280,000) over this period.

## Discussion

From 2011 to 2014, substantial increases were observed in current e-cigarette and hookah use among middle and high school students, resulting in an overall estimated total of 2.4 million e-cigarette youth users and an estimated 1.6 million hookah youth users in 2014. Statistically significant decreases occurred in the use of cigarettes, cigars, tobacco pipes, bidis, and snus. The increases in current use of e-cigarettes and hookahs offset the decreases in current use of other tobacco products, resulting in no change in overall current tobacco use among middle and high school students. In 2014, one in four high school students and one in 13 middle school students used one or more tobacco products in the last 30 days. In 2014, for the first time in NYTS, current e-cigarette use surpassed current use of every other tobacco product, including cigarettes.

These findings are subject to at least three limitations. First, data were collected only from youths who attended either public or private schools and might not be generalizable to all middle and high school-aged youth. Second, current tobacco use was estimated by including students who reported using at least one of the nine tobacco products asked in the survey but might have had missing responses to any of the other eight tobacco products; missing responses were considered as nonuse, which might have resulted in underestimated results. Finally, changes between 2013 and 2014 in the wording and placement of questions about the use of e-cigarettes, hookahs, and tobacco pipes might have had an impact on reported use of these products. Despite these limitations, overall prevalence estimates are similar to the findings of other nationally representative youth surveys (6,7).

Tobacco prevention and control strategies, including increasing tobacco product prices, adopting comprehensive smoke-free laws, and implementation of national public education media campaigns, might have influenced the reduction of cigarette smoking in youths (2). However, the lack of decline in overall tobacco use from 2011 to 2014 is concerning and indicates that an estimated 4.6 million youths continue to be exposed to harmful constituents, including nicotine, present in tobacco products (Table). Youth use of tobacco in any form, whether it be combustible, noncombustible, or electronic, is unsafe (1); regardless of mode of delivery, nicotine exposure during adolescence, a critical time for brain development, might have lasting adverse consequences for brain development (1), causes addiction (3), and might lead to sustained use of tobacco products. Rapid changes in use of traditional and emerging tobacco products among youths underscore the importance of enhanced surveillance of all tobacco use.

What is already known on this topic?

Tobacco use and addiction most often begins during youth and young adulthood. Youth use of tobacco in any form is unsafe and might have lasting adverse consequences on their developing brains.

What is added by this report?

In 2014, an estimated 4.6 million youths, including 3.7 million high school and 900,000 middle school students, reported current use (use on one or more days in the past 30 days) of any tobacco product. From 2011 to 2014, statistically significant increases were observed in e-cigarette and hookah use among high school and middle school students, while statistically significant decreases were observed in the use of cigarettes, cigars, tobacco pipes, bidis, and snus. The increases in current use of e-cigarettes and hookahs offset the decreases in other tobacco products, resulting in no change in overall current tobacco use among youths.

What are the implications for public health practice?

In 2014, nearly one in four high school students and one in 13 middle school students reported current use of any tobacco product. Because the use of emerging tobacco products (e-cigarettes and hookahs) is on the rise among middle and high school students, it is critical that comprehensive tobacco control and prevention strategies for youths should address all tobacco products and not just cigarettes.

Sustained efforts to implement proven tobacco control policies and strategies are necessary to prevent youth use of all tobacco products. In April 2014, FDA issued a proposed rule to deem all products made or derived from tobacco subject to FDA jurisdiction, and the agency is reviewing public comments on the proposed rule (8). Regulation of the manufacturing, distribution, and marketing of tobacco products coupled with full implementation of comprehensive tobacco control and prevention strategies at CDC-recommended funding levels could reduce youth tobacco use and initiation (1,2,9). Because use of emerging tobacco products (e-cigarettes and hookahs) is increasing among middle and high school students, it is critical that comprehensive tobacco control and prevention strategies for youths should address all tobacco products and not just cigarettes.

## Figures and Tables

**FIGURE 1:**
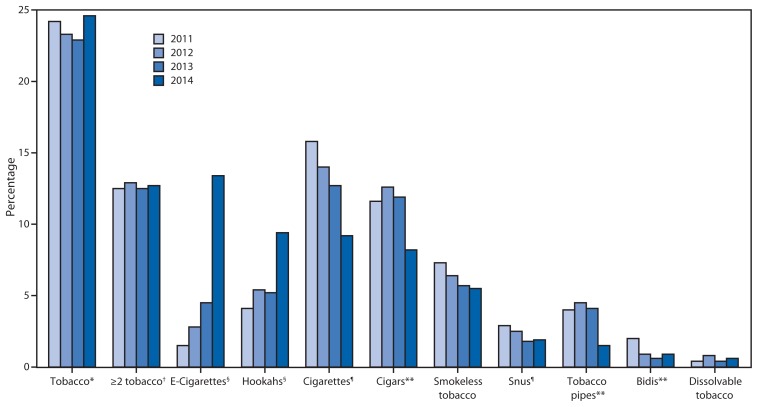
Estimated percentage of high school students who used tobacco in the preceding 30 days, by tobacco product — National Youth Tobacco Survey, United States, 2011–2014 * Defined as preceding 30-day use of cigarettes, cigars, smokeless tobacco, e-cigarettes, hookahs, tobacco pipes, snus, dissolvable tobacco, and/or bidis. ^†^Defined as preceding 30-day use of two or more of cigarettes, cigars, smokeless tobacco, e-cigarettes, hookahs, tobacco pipes, snus, dissolvable tobacco, and/or bidis. ^§^ Linear decrease (p<0.05). ^¶^ Nonlinear increase (p<0.05). ** Nonlinear decrease (p<0.05).

**FIGURE 2:**
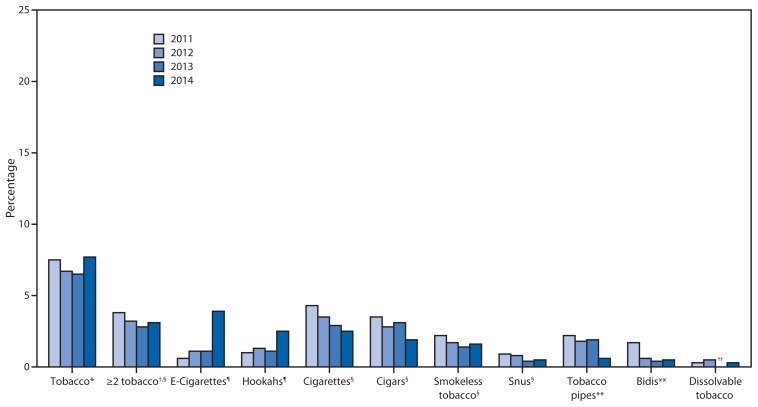
Estimated percentage of middle school students who used tobacco in the preceding 30 days, by tobacco product — National Youth Tobacco Survey, United States, 2011–2014 * Defined as preceding 30-day use of cigarettes, cigars, smokeless tobacco, e-cigarettes, hookahs, tobacco pipes, snus, dissolvable tobacco, and/or bidis. ^†^Defined as preceding 30-day use of two or more of cigarettes, cigars, smokeless tobacco, e-cigarettes, hookahs, tobacco pipes, snus, dissolvable tobacco, and/or bidis. ^§^ Linear decrease (p<0.05). ^¶^ Nonlinear increase (p<0.05). ** Nonlinear decrease (p<0.05). ^††^ Data statistically unstable.

**TABLE:** Estimated percentage of tobacco use in the preceding 30 days by product,* school level, sex, and race/ethnicity — National Youth Tobacco Survey, United States, 2014

Tobacco product	Sex				Race/Ethnicity								Total		
	
	Female		Male		Non-Hispanic White		Non-Hispanic Black		Hispanic†		Non-Hispanic other race				
						
	%	(95% CI)	%	(95% CI)	%	(95% CI)	%	(95% CI)	%	(95% CI)	%	(95% CI)	%	(95% CI)	Estimated no. of users§
**High school students**															
Electronic cigarettes	11.9	(9.7–14.5)	15.0	(12.4–18.2)	15.3	(12.4–18.8)	5.6	(3.7–8.5)	15.3	(11.8–19.5)	9.4	(6.8–12.9)	13.4	(11.2–16.1)	2,010,000
Hookah	9.8	(8.3–11.5)	8.9	(7.5–10.4)	9.4	(8.0–11.0)	5.6	(4.3–7.2)	13.0	(10.5–16.0)	6.0	(4.0–8.8)	9.4	(8.2–10.7)	1,380,000
Cigarettes	7.9	(6.8–9.1)	10.6	(9.0–12.4)	10.8	(9.3–12.5)	4.5	(3.6–5.8)	8.8	(7.2–10.7)	5.3	(3.5–7.8)	9.2	(8.1–10.4)	1,370,000
Cigars	5.5	(4.6–6.7)	10.8	(9.5–12.3)	8.3	(7.1–9.7)	8.8	(6.8–11.4)	8.0	(6.5–9.8)	2.6	(1.7–4.2)	8.2	(7.2–9.2)	1,200,000
Smokeless tobacco	1.2	(0.9–1.6)	9.9	(8.1–12.1)	7.8	(6.4–9.5)	1.1	(0.6–2.0)	3.1	(2.3–4.1)	—¶	—	5.5	(4.6–6.7)	830,000
Snus	0.8	(0.6–1.2)	3.0	(2.2–4.0)	2.4	(1.8–3.2)	0.6	(0.4–1.1)	1.5	(1.0–2.3)	—	—	1.9	(1.5–2.4)	280,000
Pipes	0.9	(0.7–1.3)	2.1	(1.6–2.9)	1.9	(1.4–2.5)	—	—	1.5	(1.0–2.2)	—	—	1.5	(1.2–2.0)	220,000
Bidis	0.6	(0.4–0.8)	1.2	(0.9–1.6)	0.8	(0.6–1.2)	—	—	1.1	(0.7–1.7)	—	—	0.9	(0.7–1.2)	130,000
Dissolvable tobacco	0.4	(0.2–0.6)	0.8	(0.5–1.1)	0.6	(0.4–0.9)	—	—	0.7	(0.4–1.2)	—	—	0.6	(0.5–0.8)	80,000
Any tobacco product use**	20.9	(18.8–23.2)	28.3	(25.6–31.1)	26.5	(23.9–29.4)	17.2	(14.8–20.0)	26.7	(23.0–30.7)	15.3	(11.5–20.1)	24.6	(22.6–26.7)	3,720,000
≥ 2 tobacco product use††	10.0	(8.6–11.6)	15.3	(13.4–17.4)	15.1	(13.3–17.1)	5.4	(4.0–7.3)	12.6	(10.5–15.1)	7.0	(4.7–10.1)	12.7	(11.2–14.3)	1,910,000
**Middle school students**															
Electronic cigarettes	3.3	(2.5–4.3)	4.5	(3.4–5.9)	3.1	(2.2–4.2)	3.8	(2.5–5.6)	6.2	(4.8–7.9)	—	—	3.9	(3.0–5.0)	450,000
Hookah	2.6	(1.9–3.5)	2.4	(1.9–3.0)	1.4	(1.1–1.9)	—	—	5.6	(4.4–7.1)	—	—	2.5	(2.0–3.0)	280,000
Cigarettes	2.0	(1.5–2.6)	3.0	(2.3–3.9)	2.2	(1.6–3.1)	1.7	(1.1–2.9)	3.7	(2.7–5.1)	—	—	2.5	(2.1–3.0)	290,000
Cigars	1.4	(1.0–2.1)	2.4	(1.7–3.5)	1.4	(0.9–2.4)	2.0	(1.3–2.9)	2.9	(2.2–3.8)	—	—	1.9	(1.5–2.5)	220,000
Smokeless tobacco	—	—	2.1	(1.4–3.1)	1.7	(1.1–2.6)	—	—	1.3	(0.9–2.0)	2.4	(1.4–4.1)	1.6	(1.2–2.2)	180,000
Snus	—	—	0.7	(0.4–1.2)	—	—	—	—	—	—	—	—	0.5	(0.3–0.8)	50,000
Pipes	—	—	0.6	(0.4–0.9)	0.5	(0.3–0.8)	—	—	0.9	(0.6–1.4)	—	—	0.6	(0.4–0.8)	60,000
Bidis	0.3	(0.2–0.5)	—	—	—	—	—	—	0.6	(0.4–0.9)	—	—	0.5	(0.3–0.9)	60,000
Dissolvable tobacco	—	—	0.4	(0.2–0.6)	—	—	—	—	—	—	—	—	0.3	(0.1–0.5)	30,000
Any tobacco product use	6.6	(5.4–8.1)	8.8	(7.6–10.1)	6.2	(5.1–7.4)	7.3	(5.6–9.3)	11.8	(9.9–14.1)	6.4	(4.1–9.9)	7.7	(6.7–8.9)	910,000
≥2 tobacco product use	2.4	(1.8–3.1)	3.8	(3.0–4.7)	2.5	(1.9–3.3)	2.0	(1.3–3.2)	5.0	(4.2–5.9)	—	—	3.1	(2.6–3.7)	360,000

**Abbreviation:** CI = confidence interval

* Preceding 30-day use of cigarettes was determined by asking, “During the past 30 days, on how many days did you smoke cigarettes?”; preceding 30-day use of cigars was determined by asking, “During the past 30 days, on how many days did you smoke cigars, cigarillos, or little cigars?”; preceding 30 day use of smokeless tobacco was determined by asking, “During the past 30 days, on how many days did you use chewing tobacco, snuff, or dip?”; preceding 30-day use of electronic cigarettes was determined by asking, “During the past 30 days, on how many days did you use electronic cigarettes or e-cigarettes such as Blu, 21st Century Smoke, or NJOY?”; preceding 30-day use of hookahs, pipe (not hookah), snus, dissolvable tobacco, and bidis was determined by asking, “In the past 30 days, which of the following products have you used on at least 1 day?”

† Persons of Hispanic ethnicity can be of any race or combination of races.

§ Estimated total number of users is rounded down to the nearest 10,000.

¶ Data are statistically unreliable because sample size was <50 or relative standard error was >0.3.

** Defined as preceding 30-day use of cigarettes, cigars, smokeless tobacco, electronic cigarettes, hookahs, tobacco pipes, snus, dissolvable tobacco, and/or bidis on ≥1 day in the past 30 days.

†† Defined as preceding 30-day use of two or more of cigarettes, cigars, smokeless tobacco, electronic cigarettes, hookahs, tobacco pipes, snus, dissolvable tobacco, and/or bidis on ≥1 day in the past 30 days.

## Footnotes

* Kreteks no longer are sold legally in the United States and therefore data on these products were not collected in the 2014 cycle of NYTS. Kreteks also were not included in the definition of tobacco in years (2011, 2012, and 2013) in which the data were collected in order to be enable researchers to assess trends across the study period.

† In 2014, current use of e-cigarettes was assessed by the question, “During the past 30 days, on how many days did you use e-cigarettes such as Blu, 21st Century Smoke, or NJOY?”, and in 2011 to 2013, such use was assessed by the question, “In the past 30 days, which [tobacco products] have you used on at least 1 day?”

§ In 2014, current use of hookahs was assessed by the question, “In the past 30 days, which [tobacco products] have you used on at least one day?” and was the first response option available to be selected; whereas from 2011 to 2013, hookah was the fourth or fifth response option.

¶ In 2014, current use of tobacco pipes was assessed by the question, “In the past 30 days, which [tobacco products] have you used on at least 1 day?” and in 2011 to 2013, it was assessed by the question, “During the past 30 days, on how many days did you smoke tobacco in a pipe?”

** A test for linear trend is significant if an overall statistically significant decrease or increase occurs during the study period. Data also were assessed for the presence of nonlinear trends; a significant nonlinear trend indicates that the rate of change changed across the study period.

†† Persons of Hispanic ethnicity can be of any race or combination of races.
