# Errata

**Published:** 2015-04-24

## Vol. 64, No. 14

In the report, “Tobacco Use Among Middle and High School Students — United States, 2011–2014,” errors occurred in the third and fourth footnotes to Figure 1 on page 383. Those footnotes should read as follows:

^§^ Nonlinear increase (p<0.05).

^¶^ Linear decrease (p<0.05).

